# Clinical influence of switching companion diagnostic tests for EGFR‐TKs from Therascreen to Cobas v2

**DOI:** 10.1111/1759-7714.13797

**Published:** 2021-02-02

**Authors:** Ken Uchibori, Natsuki Takano, Ryo Manabe, Ryosuke Tsugitomi, Shinsuke Ogusu, Takehiro Tozuka, Hiroaki Sakamoto, Hiroshi Yoshida, Yoshiaki Amino, Ryo Ariyasu, Satoru Kitazono, Noriko Yanagitani, Makoto Nishio

**Affiliations:** ^1^ Department of Thoracic Medical Oncology The Cancer Institute Hospital of Japanese Foundation for Cancer Research Tokyo Japan; ^2^ Department of Pulmonary Medicine and Oncology, Graduate School of Medicine Nippon Medical School Tokyo Japan

**Keywords:** Companion diagnostic (CDx), *EGFR* mutation, EGFR‐TKI, next‐generation sequencing (NGS), polymerase chain reaction (PCR)

## Abstract

**Background:**

Several companion diagnostic (CDx) tests for epidermal growth factor receptor tyrosine kinase inhibitors (EGFR‐TKIs) have been approved. In our institute, the CDx test for EGFR‐TKIs was changed from the Therascreen test (Therascreen) to the Cobas EGFR v2 test (Cobas) because only Cobas was approved for the use of osimertinib in patients with *EGFR*‐mutated non‐small cell lung cancer (NSCLC) with T790M mutations. The clinical influence of switching the CDx test has not yet been examined comprehensively.

**Methods:**

All serial patients with lung cancer tested for *EGFR* mutations with CDx tests between February 2014 and February 2016 at the Cancer Institute Hospital of the Japanese Foundation for Cancer Research (JFCR) were enrolled in this analysis.

**Results:**

Therascreen was used as a CDx test for EGFR‐TKI therapy in 607 patients between February 2014 and January 2015, and Cobas was used in 621 patients between February 2015 and February 2016. *EGFR* mutations were detected in 218 patients (35.9%) and 244 patients (39.3%) tested with Therascreen and Cobas, respectively. At the initial diagnosis, 400 and 459 patients were tested with Therascreen and Cobas, respectively. *EGFR* mutation subtypes, including del19, L858R, and others, were detected in 13.0%, 17.0%, and 2.5% of patients using Therascreen and 17.4%, 14.4%, and 1.5% of patients using Cobas, respectively.

**Conclusions:**

No significant impact of switching from Therascreen to Cobas as the CDx test for *EGFR* mutations in clinical practice was observed. However, the detection pattern of the *EGFR* mutation subtypes between the two CDx tests was slightly different.

**Key points:**

**Significant findings of the study:**

We examined the influence of changing the EGFR test in 1228 patients in total. The detection rate of *EGFR* mutations was similar. However, the detection pattern for *EGFR* subtype mutations was slightly different between the two tests.

**What this study adds:**

Switching CDx tests from target polymerase chain reaction (PCR)‐ to next‐generation sequencing (NGS)‐based methods may lead to obvious changes in clinical practice. When the CDx test is required to change, the investigation of this influence is warranted in future studies.

## Graphical abstract

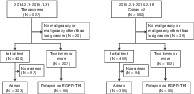

We examined the influence of changing the EGFR test among target PCR methods in 1228 patients in total. The detection rate of *EGFR* mutations was similar while the detection pattern for *EGFR* subtype mutations was slightly different between the two tests. In the future, switching companion diagnostic (CDx) tests from target PCR methods to NGS‐based methods may lead to obvious changes in clinical practice.

## Introduction

Epidermal growth factor receptor (*EGFR*) gene‐activating mutations are the primary oncogenic drivers in lung adenocarcinoma.^1^ The overall survival of patients with *EGFR*‐mutated lung cancer has improved remarkably from one year to approximately three to four years after the introduction of EGFR tyrosine kinase inhibitors (EGFR‐TKIs).^2^, ^3^, ^4^, ^5^, ^6^, ^7^ The detection of *EGFR*‐activating mutations using companion diagnostic (CDx) tests is mandatory to initiate treatment with EGFR‐TKIs. Variable EGFR tests are used in clinical practice in Japan. Therascreen,^8^, ^9^, ^10^ Cobas EGFR v2,^11^, ^12^ and Oncomine CDx^13^, ^14^ are the CDx tests used for EGFR therapy that were approved in 2011, 2016, and 2019, respectively. These CDx tests each have a unique profile in terms of the detection method and degree of specificity for *EGFR* gene mutations (Table S1), especially *EGFR* exon 19 deletions (del19), which have been shown to have many patterns in DNA sequences, leading to the identification of ~30 types.^15^

Therascreen (Therascreen) can detect *EGFR* gene mutations using a polymerase chain reaction (PCR)‐based Scorpion‐ARMS method that covers three types of G719X, 19 types of del19, three types of exon 20 ins, S768I, T790M, L858R, and L861Q, with a detection sensitivity of 1%–10%. Cobas EGFR v2 (Cabas) can detect *EGFR* gene mutations using a PCR‐based Cobas method that covers three types of G719X (exon 18), 29 types of del19, five types of exon 20 ins, S768I, T790M, two types of L858R, and L861Q, with a detection sensitivity of 3%–5%. Importantly, Cobas is the only CDx test for T790M mutations to prescribe osimertinib. Oncomine CDx (Oncomine) was the first approved CDx test based on next‐generation sequencing (NGS) in Japan, and it targets multiple oncogenes including *EGFR, ALK, ROS1*, and *BRAF*. The profile of *EGFR* mutations detected by Oncomine includes four types of G719X (exon 18), 21 types of del19, three types of exon 20 ins, S768I, T790M, two types of L858R, L861Q, and L861R, with a detection sensitivity of 6%–8%.

The results of testing *EGFR* mutations might depend on which CDx test is used, and it is essential to determine the influence of switching the CDx test in clinical practice. In our institute, we were evaluating *EGFR* mutations with Therascreen, which was approved as a CDx test for EGFR‐TKIs in January 2013. In February 2015, we switched from Therascreen to EGFR‐Cobas for *EGFR* mutation testing when osimertinib was approved for the treatment of patients with T790M‐positive lung cancer who relapsed on prior EGFR‐TKI therapy because only Cobas was approved as a CDx test for the use of osimertinib in patients with T790M mutations. The details of detectable del19 mutations are different between Therascreen and Cobas. Therascreen and Cobas do not cover all types of del19 mutations, and there are some differences in the mutations covered by these tests, as shown in the package inserts. Detectable uncommon mutations, such as G719X and ex 20 insertions, are more relevant in testing with Cobas than with Therascreen. The main objective of this study was to compare the frequency of *EGFR* mutations and the distribution of mutation subtypes after the replacement of Therascreen with Cobas.

## Methods

We analyzed all patients who were tested for *EGFR* mutations between February 2014 and February 2016 at the Cancer Institute Hospital of the Japanese Foundation for Cancer Research (JFCR). Therascreen was used to detect *EGFR* mutations from February 2014 to January 2015, and Cobas was used to detect *EGFR* mutations from February 2015 to February 2016. Cytology samples or tissue samples were obtained by transbronchial biopsy, computed tomography (CT)–guided biopsy, and surgery. All samples were analyzed at the CLIA certified commercial laboratories SRL and LSI using Therascreen and Cobas, respectively.

We evaluated the frequency of each *EGFR* mutation type, including activating mutations (exon 19 deletion [del19] and exon 21 point mutations [L858R]), uncommon mutations (G719X, S768I, L861Q/R, and exon 20 ins), and T790M, according to the detection methods Therascreen and Cobas using a subgroup analysis involving all participants, overall patients at the initial diagnosis, adeno/nonadeno subtypes at the initial diagnosis, and rebiopsy at failure on EGFR‐TKIs. The difference in background characteristics and the detection frequencies of subtypes between the two groups was calculated statistically using the chi‐squared test. Differences in the median age were analyzed with the Mann‐Whitney U test. The GraphPad prism 7 (GraphPad Software, San Diego, CA) was used for statistical analysis. Differences were considered statistically significant at *P* < 0.05.

The study protocol was reviewed and approved by the Ethics Committee of the Cancer Institute Hospital, JFCR (IRB number: 2020–1139).

## Results

### Patient characteristics

**Figure 1:**
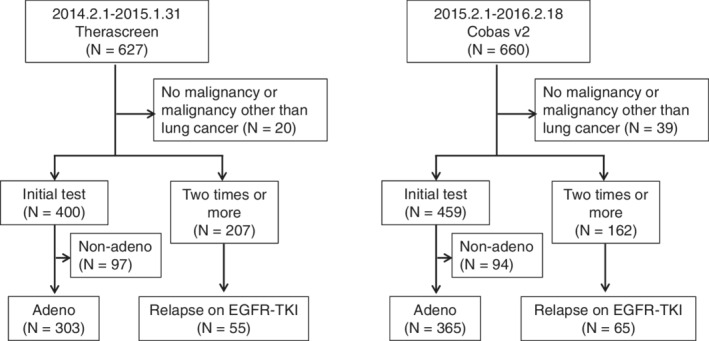
Patient flow diagram. Adeno, adenocarcinoma; Initial test, EGFR test at the initial attempt; Nonadeno, histology other than adenocarcinoma; ≥2 times, EGFR test at the second attempt or over.

Of 1287 patients who underwent *EGFR* mutation tests, 607 patients were examined with Therascreen and 621 patients were examined with Cobas. The remaining 59 patients were excluded because they had malignancies other than lung cancer (Fig 1). The patient characteristics of all tested cases are shown in Table 1. A few cases were doubly counted because the plural time of tests was examined for these cases in the study period. The patients' age and the timing of testing were statistically different between the two groups, whereas other factors, including sex, smoking history, pathology, stage, PS, and tested specimens, were similar.

**Table 1:** Patient characteristics

		Therascreen (*N* = 607)	Cobas (*N* = 621)	*P* (χ^2^)
Age	Median (range)	67 (19–90)	69 (38–92)	0.017
Sex	Male/female	335/272	334/287	0.352
Smoking history	Never/ex or current/unknown	217/390/0	251/369/1	0.143
Pathology	Adeno/nonadeno	482/125	506/115	0.359
Stage	I–III/IV or recurrence	360/247	392/229	0.317
PS (ECOG)	0,1/≥2	576/31	581/40	0.170
Timing of test	Initial/2nd or more	400/207	459/162	0.002
Timing of test	Post‐EGFR‐TKI	55	66	0.362
Tested specimens	Cytology/histology	264/343	300/321	0.090
EGFR status	Wild/positive/not detected	378/218/11	367/244/10	0.469
	Wild	378	367	0.255
	Ex19 del	94	118	0.103
	Ex21 L858R	105	110	0.848
	T790M (de novo/acquired)	22 (1/21)	20 (0/20)	0.697
	Other	G179X‐3	G719X‐3	0.600
		S768I‐3	G719X + S768I‐1	
		Ex20 ins‐6	G719X + T790M‐2	
		L861Q‐4	Ex20 ins‐7	
		L858R + S768I‐1	L861Q‐0	
		L858R + Ex20 ins‐1	L858R + S768I‐3	
		L858R + del19–1		
	Not detected	11	10	0.785

Adeno, adenocarcinoma; Ex19 del, EGFR exon 19 deletion; Ex20 ins, EGFR exon 20 insertion; Ex21 L858R, EGFR exon 21 L858R point mutation; ex or current, ex or current smoker; initial, initial attempt of EGFR test; Never, never smoked; nonadeno, pathology other than adenocarcinoma; not detected: the result was not detected with EGFR test; positive, *EGFR* mutation‐positive; wild, wild‐type; second or more, EGFR test at second (or more) times.

The detection rate of common *EGFR* mutations (del19 and L858R) was 32.8% in the Therascreen group and 36.7% in the Cobas group, whereas that of uncommon mutations, such as G719X and exon20 ins, was 3.1% and 2.6%, respectively. T790M mutations were detected in 3.6% and 3.2% of all tested patients in the Therascreen and Cobas groups, respectively. Only one case was a de novo T790M mutation occurring at the initial diagnosis. T790M cases were not added to the EGFR‐positive cases because they were already counted as del19 or L858R‐positive cases. The prevalence of *EGFR* mutations between the two groups did not differ significantly.

### Patient characteristics at the initial test

Focusing on the patients whose EGFR status was evaluated at the initial test, 400 patients were examined by Therascreen and 459 patients were analyzed by Cobas (Table 2). The characteristics were similar between the two groups, with slight differences in age, pathology, and tested specimens. The proportion of adenocarcinoma was dominant in each group (75.8% in Therascreen and 79.5% in Cobas).

**Table 2:** Characteristics of all patients at the initial EGFR test

		Therascreen (*N* = 400)	Cobas (*N* = 459)	*P*‐value (χ^2^)
Age	Median (range)	67 (29–90)	69 (38–92)	0.216
Sex	Male/female	232/168	261/198	0.737
Smoking history	Never/ex or current/unknown	138/262/0	179/279/1	0.247
Pathology	Adeno/nonadeno	303/97	365/94	0.185
Stage	I–III/IV or recurrence	291/109	332/127	0.891
PS (ECOG)	0,1/≥2	386/14	437/22	0.346
Tested specimens	Cytology/histology	187/213	214/245	0.970
EGFR status	Wild/positive/not detected	265/130/5	300/153/6	0.963
	Wild	265	300	0.784
	Ex19 del	52 (+T790M 1)	80	0.073
	Ex21 L858R	68	66	0.291
	T790M	1	0	0.795
	Other	G719X‐1 S768I‐2 Ex20 ins‐4 L861Q‐2 L858R+S768I‐1	G719X‐1 G719X+S768I‐1 Ex20 ins‐3 L861Q‐0 L858R+S768I‐2	0.306
	Not detected	5	6	0.957

Adeno, adenocarcinoma; Ex19 del, EGFR exon 19 deletion; Ex20 ins, EGFR exon 20 insertion; Ex21 L858R, EGFR exon 21 L858R point mutation; ex or current, ex or current smoker; Never, never smoked; nonadeno, pathology other than adenocarcinoma; not detected, the result was not detected with EGFR test; positive, *EGFR* mutation‐positive; wild, wild‐type.

The proportion of del19, L858R, ex20 ins, and other minor mutations was 13.0%, 17.0%, 1.0%, and 1.5% in the Therascreen group and 17.4%, 14.4%, 0.7%, and 0.9% in the Cobas group, respectively. A de novo T790M mutation was found in only one case (0.25%) in the Therascreen group. The frequencies of del19 and L858R among *EGFR* mutations were 40% and 52.3% for Therascreen and 52.3% and 43.1% for Cobas, respectively.

In contrast to our expectations, del19 mutations were observed numerically but not significantly more frequently (*P* = 0.073) in the Cobas group compared with the Therascreen group, whereas the prevalence of L858R was slightly higher in the Therascreen group than in the Cobas group but without statistical significance (*P* = 0.291). The distribution of uncommon mutations was not different between the two groups (*P* = 0.306).

### Patients with adenocarcinoma at the initial test

From the perspective of detection performance regarding common *EGFR* mutations in lung adenocarcinoma at the initial EGFR testing, our results showed that the detection rates of overall mutations and common mutations (del19 and L858R) were similar (42.2% vs. 41.9% and 39.3% vs. 40.0% for Therascreen and Cobas, respectively) (Table 3). The frequencies of del19 and L858R among *EGFR* mutations were 39.8% and 53.1% in the Therascreen group and 52.3% and 43.1% in the Cobas group, respectively. Similar to the analysis of all participants, del19 mutations in the Cobas group and L858R mutations in the Therascreen group were more prevalent numerically but not statistically significant than those in each other's group (*P* = 0.099 and 0.161, respectively). The distribution of uncommon mutations was not different between the two groups (*P* = 0.378).

**Table 3:** Patient characteristics at the initial EGFR test divided into pathological types

		Adeno			Nonadeno		
		Therascreen (*N* = 303)	Cobas (*N* = 365)	*P*‐value (χ^2^)	Therascreen (*N* = 97)	Cobas (*N* = 94)	*P*‐value (χ^2^)
Age	Median (range)	66 (29–90)	68 (38–92)	0.07	71 (43–87)	71 (38–85)	0.679
Sex	Male/female	160/143	185/180	0.585	72/25	76/18	0.273
Smoking history	Never/ex or current/unknown	128/175/0	172/192/1	0.285	10/87	7/87	0.487
Stage	I–III/IV or recurrence	221/82	272/93	0.643	70/27	60/34	0.217
PS (ECOG)	0,1/≥2	295/8	349/16	0.228	91/6	88/6	0.955
Tested specimens	Cytology/histology	125/178	152/213	0.919	62/35	62/32	0.768
EGFR status	Wild/positive/not detected	174/128/3	207/153/5	0.899	93/2/2	93/0/1	0.319
	Wild	174	207	0.853	93	93	0.853
	Ex19 del	51 (+T790M 1)	80	0.099	1	0	0.326
	Ex21 L858R	68	66	0.161	0	0	—
	T790M	1	0	0.272	0	0	—
	Other	G719X‐1 S768I‐1 Ex20 ins‐4 L861Q‐2 L858R + S768I‐1	G719X‐1 G719X + S768I‐1 Ex20 ins‐3 L861Q‐0 L858R + S768I‐2	0.378	G719X‐0 S768I‐1 Ex20 ins‐0	G719X‐0 S768I‐0 Ex20 ins‐0	0.323
	Not detected	3	5	0.653	2	1	0.579

Adeno, adenocarcinoma; Ex19 del, EGFR exon 19 deletion; Ex20 ins, EGFR exon 20 insertion; Ex21 L858R, EGFR exon 21 L858R point mutation; ex or current, ex or current smoker; Never, never smoked; nonadeno, pathology other than adenocarcinoma; not detected, the result was not detected with EGFR test; positive, *EGFR* mutation‐positive; wild, wild‐type.

In the nonadeno group, there were no differences in the background characteristics of patients included for each detection method. Only one del19 and one S768I *EGFR* mutation in the Therascreen group were confirmed in this cohort.

### Details of rebiopsies at relapse on EGFR‐TKIs

A rebiopsy is usually performed at failure on first‐ or second‐generation EGFR‐TKI therapy to evaluate the development of T790M secondary mutations. In this study, 55 and 66 cases were assessed by Therascreen and Cobas, respectively. The characteristics of patients who underwent a rebiopsy were generally similar in the clinic, although seven cases with histology results other than adenocarcinoma or squamous cell carcinoma and predominance in cytology specimens among the evaluated samples were observed in the Cobas group (Table 4).

**Table 4:** Patient characteristics who underwent rebiopsy at relapse on EGFR‐TKIs

		Therascreen (*N* = 55)		Cobas (*N* = 65)		*P*‐value (χ^2^)
Age	Median (range)	65 (34–83)		67 (40–86)		0.130
Sex	Male/female	15/40		24/41		0.261
Smoking history	Never/ex or current/unknown	31/24/0		29/36/0		0.200
Pathology	Adeno/nonadeno	55/0		59/6		0.021
PS(ECOG)	0, 1/≥2	48/7		51/4		0.340
Tested specimens	Cytology/histology	39/16		58/7		0.011
EGFR status	Wild/positive/not detected	3/48/4		10/53/2		0.144
	Wild	3		10		0.081
	Ex19 del	10		12		0.969
	Ex21 L858R	15		17		0.890
	T790M	G179X + T790M	0	G179X + T790M	2	0.318
		Del19 + T790ML8	15	Del19 + T790M	11	
		58R + T790M	4	L858R + T790M	7	
	Other	G719X	1	G719X	1	0.
		L858R + Ex20ins	1	Ex20ins	3	
		S768I	0	S768I	0	
		L861Q	1	L861Q	0	
		L858R + del19	1			
	Not detected	4		2		0.293

Adeno, adenocarcinoma; ex or current: ex or current smoker; Ex19 del, EGFR exon 19 deletion; Ex21 L858R, EGFR exon 21 L858R point mutation; Ex20 ins, EGFR exon 20 insertion; Never, never smoked; nonadeno, pathology other than adenocarcinoma; not detected, the result was not detected with EGFR test; positive, *EGFR* mutation‐positive; wild, wild‐type.

The comparison of the detection sensitivity for T790M mutations revealed no significant difference between Therascreen (34.5% [19/55]) and Cobas (30.8% [20/65]) (*P* = 0.762). The proportions of T790M‐negative, wild‐type, and undetected tumors were similar between the groups (45.5% vs. 44.6%, 5.5% vs. 15.4%, and 7.3% vs. 3.1% for Therascreen and Cobas, respectively). The informative rebiopsy rates in this study estimated by excluding the wild‐type and undetected tumors were 87.7% (48/55) and 81.5% (53/65) for Therascreen and Cobas, respectively. The detection power of T790M in informative biopsies was 39.6% for Therascreen and 37.7% for Cobas. These results suggest that the performance of the two methods is not significantly different.

## Discussion

We evaluated the *EGFR* mutation status for the treatment of lung cancer according to the results of approved CDx tests to prescribe EGFR‐TKIs. However, we usually use only one test in each institution. To the best of our knowledge, there is no report other than the current study that evaluates the influence of replacing the CDx test in clinical practice, but some reports have compared Scorpion and Cobas using identical specimens.^16^, ^17^, ^18^, ^19^, ^20^

As a result, no significant difference between the characteristics of patients tested with the two CDx tests was observed, except for age. The frequency of *EGFR* mutations and the distribution of mutation subtypes did not differ significantly before and after the replacement of Therascreen with Cobas. Our results show that the frequency of *EGFR* mutations at the initial testing among all patients and those with adenocarcinoma was 32.5% versus 33.3% and 42.2% versus 41.9% for Therascreen versus Cobas, respectively. The frequencies of del19 and L858R among *EGFR* mutations in adenocarcinoma were 39.8% and 53.1% in the Therascreen group and 52.3% and 43.1% in the Cobas group, respectively. These results were similar to previous reports showing that *EGFR* mutations account for 30%–40% of non‐small cell lung cancer (NSCLC) and 40%–55% of lung adenocarcinoma and that del19 mutations account for 40%–49% and 46%–59%, respectively, whereas L858R mutations account for 39%–47% and 25%–38% of *EGFR* mutations among Asians and Caucasians, respectively.^21^, ^22^, ^23^, ^24^ The frequency of uncommon mutations among *EGFR* mutation‐positive adenocarcinoma cases (7.0% [9/128] in Therascreen and 4.6% [7/153] in Cobas) was not significantly different. It was difficult to compare the details because of the small number of cases. Only two EGFR‐positive cases were confirmed among the nonadenocarcinoma cohort, supporting the established knowledge that *EGFR* mutations are rarely found in nonadenocarcinoma histology.^25^, ^26^

At the initial diagnosis test, we anticipated that more del19 cases might be found in the Cobas group than in the Therascreen group based on the wider coverage of Cobas for detectable del19 types. As we expected, the number of del19 mutations in the current study was larger in the Cobas group than in the Therascreen group, although this difference was not statistically significant. On the other hand, the distribution of L858R among *EGFR* mutation‐positive cases was reduced to 42.3% for Cobas from 53.1% in the Therascreen group, with no statistical significance. These disparities between the two groups in the detection rate of *EGFR*‐activating mutations and the distribution of mutation subtypes stayed within the range mentioned above, suggesting that the replacement of Therascreen with Cobas has minimal influence in clinical practice. One of the reasons that switching the CDx test did not influence the detection rate in clinical practice was that both methods are mutation‐specific target PCR‐based CDx tests.

As cytological specimens are acceptable for PCR‐based EGFR testing,^27^ about half of the samples assessed were cytological specimens (46.8% for Therascreen and 46.6% for Cobas). The ratio of cytological samples was similar between the two groups. The combined use of cytological and histological samples did not affect the detection rate of *EGFR* mutations for either Therascreen or Cobas. Regarding the perspective of testing at failure on EGFR‐TKIs, we should note that cytological samples were more prevalent in the current study than those in previous studies.^28^, ^29^ In this study, the detection power of T790M mutations in informative biopsies was 39.6% for Therascreen and 37.7% for Cobas, which are slightly lower than those reported previously. However, the performance of the two tests was similar.

Recently, NGS‐based CDx tests, such as the Oncomine CDx System and FoudationOne, have been approved for EGFR‐TKIs and other molecular agents. The method used to evaluate driver oncogenes in lung cancer has been switched gradually from single‐plex mutation‐specific target PCR CDx tests, such as Cobas or Therascreen, to multi‐plex CDx tests based on NGS. Clinical properties between PCR‐ and NGS‐based tests may be quite different. Therefore, we should pay attention to the influence of switching from target PCR‐ to NGS‐based CDx tests, which might lead to obvious changes in clinical practice.

Some limitations of this study should be addressed. First, this is a retrospective study from a single institution comparing two distinct patient groups. Second, the EGFR detection profiles of Therascreen and Cobas were not compared directly by assessing identical specimens. The results of this study might include selection bias. The concordance rate between Therascreen and Cobas was reported to be 98.0% when they were evaluated using identical specimens.^30^ However, these points may be permitted because the primary focus of this study was to investigate the influence of switching the EGFR test in clinical practice. We considered it preferable to compare sequential patients in a year at a single institution rather than at multiple institutions. The consistency in sample collection and handling procedures might provide advantages for this study to evaluate the clinical properties of CDx tests. Moreover, the results of this study might reflect the quality of CDx tests in the real world because variable sample sizes and samples obtained in practice were assessed, whereas a usual performance test is conducted using samples of ideal size and quality. Taken together, the results of this study provide useful information to examine the influence of switching the CDx test in clinical practice.

In conclusion, the switching of CDx tests from Therascreen to Cobas showed minimal influence in clinical practice, but the distribution pattern of *EGFR* mutation subtypes might differ. Switching CDx tests from target PCR‐ to NGS‐based methods may lead to obvious changes in clinical practice. The investigation of this influence is warranted in future studies.

## Disclosure

K. Uchibori reports the employment of a family member at Daiichi Sankyo. M. Nishio reports honoraria from Ono Pharmaceutical, Bristol‐Myers Squibb, Pfizer, Chugai Pharmaceutical, Eli Lilly, Taiho Pharmaceutical, AstraZeneca, Boehringer‐Ingelheim, MSD, and Novartis, research funding from Novartis, Daiichi Sankyo, Taiho Pharmaceutical, Bristol‐Myers Squibb, Boehringer‐Ingelheim, Ono Pharmaceutical, Eli Lilly, Chugai Pharmaceutical, AstraZeneca, Merck Serono, MSD, and Pfizer. N. Yanagitani reports honoraria from Chugai Pharmaceutical. All the other authors have stated that they have no conflicts of interest.

## Supporting information


**Table S1** The profile of detectable EGFR mutation by Cobas V2, Therascreen and Oncomine.
