# Supplementary material for: Clinical influence of switching companion diagnostic tests for EGFR‐TKs from Therascreen to Cobas v2
Source: Thorac Cancer. 2021 Feb 2;12(6):906–13. doi: 10.1111/1759-7714.13797 (PMC7952781; doi:10.1111/1759-7714.13797)
Supplement: Supplementary file 1 — Table S1 The profile of detectable EGFR mutation by Cobas V2, Therascreen and Oncomine. [file TCA-12-906-s001.docx]

**Supplementary Table**

| **exon** | **amino acid change** | **Cobas V2 [LOD (%)]** | | **Therascreen [LOD (%)]** | | **Oncomine** | **COSMIC ID** | **base change** |
| --- | --- | --- | --- | --- | --- | --- | --- | --- |
| exon3 | p.R108G |  |  |  |  | 〇 | COSM6939064 | c.322A>G |
|  | p.R108K |  |  |  |  | 〇 | COSM5576118 | c.323G>A |
| exon7 | p.A289T |  |  |  |  | 〇 | COSM6356510 | c.865G>A |
|  | p.A289D |  |  |  |  | 〇 | COSM21685 | c.866C>A |
|  | p.A289V |  |  |  |  | 〇 | COSM21687 | c.866C>T |
| exon14 | p.S492R |  |  |  |  | 〇 | COSM236671 | c.1474A>C |
|  | p.S492R |  |  |  |  | 〇 | COSM236670 | c.1476C>A |
| exon15 | p.G598V |  |  |  |  | 〇 | COSM21690 | c.1793G>T |
|  | p.G598A |  |  |  |  | 〇 | COSM3412196 | c.1793G>C |
| exon18 | p.E709K |  |  |  |  | 〇 | COSM12988 | c.2125G>A |
|  | p.E709V |  |  |  |  | 〇 | COSM12371 | c.2126A>T |
|  | p.E709G |  |  |  |  | 〇 | COSM13009 | c.2126A>G |
|  | p.E709A |  |  |  |  | 〇 | COSM13427 | c.2126A>C |
|  | p.G719C | 〇 | [5.6] | 〇 | [10.3] | 〇 | COSM6253 | c.2155G>T |
|  | p.G719S | 〇 | [3.2] | 〇 | [5.08] | 〇 | COSM6252 | c.2155G>A |
|  | p.G719A | 〇 | [2.5] | 〇 | [7.41] | 〇 | COSM6239 | c.2156G>C |
|  | p.G719D |  |  |  |  | 〇 | COSM18425 | c.2156G>A |
| exon19 | p.K745_E749del | 〇 |  |  |  | 〇 | COSM26038 | c.2233_2247del |
|  | p.K745_A750delins |  |  |  |  | 〇 | COSM1190791 | c.2234_2248del |
|  | p.E746_E749del |  |  |  |  | 〇 | COSM28517 | c.2235_2246del |
|  | .E746_A750delinsIP | 〇 |  |  |  |  | COSM13550 | c.2235_2248delinsAATTC |
|  | p.E746_A750del | 〇 | [1.4] | 〇 | [6.40] | 〇 | COSM6223 | c.2235_2249del |
|  | .E746_T751delinsIP | 〇 |  |  |  |  | COSM13552 | c.2235_2251delinsAATTC |
|  | p.E746_T751delinsI | 〇 |  | 〇 | [4.24] | 〇 | COSM13551 | c.2235_2252delinsAAT |
|  | p.E746_S752delinsI | 〇 |  |  |  |  | COSM12385 | c.2235_2255delinsAAT |
|  | p.E746_A750del | 〇 | [2.5] | 〇 | [2.80] | 〇 | COSM6225 | c.2236_2250del |
|  | p.E746_T751del | 〇 |  | 〇 | [2.43] | 〇 | COSM12728 | c.2236_2253del |
|  | p.E746_T751delinsA | 〇 |  | 〇 | [0.55] | 〇 | COSM12678 | c.2237_2251del |
|  | p.E746_T751delinsV | 〇 |  |  |  |  | COSM12386 | c.2237_2252delinsT |
|  | p.E746_T751delinsVA | 〇 | [6.3] |  |  | 〇 | COSM12416 | c.2237_2253delinsTTGCT |
|  | p.E746_S752delinsA | 〇 |  | 〇 | [2.72] |  | COSM12367 | c.2237_2254del |
|  | p.E746_S752delinsV | 〇 | [4.1] | 〇 | [1.58] | 〇 | COSM12384 | c.2237_2255delinsT |
|  | p.E746_P753delinsVS | 〇 |  |  |  |  | COSM18427 | c.2237_2257delinsTCT |
|  | p.E746_S752delinsD | 〇 |  | 〇 | [2.7] | 〇 | COSM6220 | c.2238_2255del |
|  | p.L747_A750delinsP | 〇 |  | 〇 | [3.24] | 〇 | COSM12422 | c.2238_2248delinsGC |
|  | p.L747_T751delinsQ | 〇 |  | 〇 | [16.87] | 〇 | COSM12419 | c.2238_2252delinsGCA |
|  | p.L747_T751del | 〇 | [7.2] |  |  |  | COSM12369 | c.2240_2254del |
|  | p.L747_E749del | 〇 |  | 〇 |  | 〇 | COSM144206 | c.2239_2247del |
|  | p.L747_A750delinsP | 〇 | [2.2] | 〇 | [0.25] | 〇 | COSM12382 | c.2239_2248delinsC |
|  | p.L747_T751delinsP | 〇 |  | 〇 | [4.58] | 〇 | COSM12383 | c.2239_2251delinsC |
|  | p.L747_T751del | 〇 |  | 〇 | [4.94] |  | COSM12369 | c.2239_2253del |
|  | p.L747_S752delinsQ | 〇 |  |  |  |  | COSM12403 | c.2239_2256delinsCAA |
|  | p.L747_S752del | 〇 | [4.74] | 〇 | [0.14] | 〇 | COSM6255 | c.2239_2256del |
|  | p.L747_P753delinsQ | 〇 |  | 〇 | [4.91] | 〇 | COSM12387 | c.2239_2258delinsCA |
|  | p.L747_T751delinsS | 〇 |  | 〇 | [4.09] | 〇 | COSM6210 | c.2240_2251del |
|  | p.L747_T751del | 〇 |  | 〇 |  | 〇 | COSM12369 | c.2240_2254del |
|  | p.L747_P753delinsS | 〇 | [13.4] | 〇 | [8.1] | 〇 | COSM12370 | c.2240_2257del |
|  | p.S752_I759del | 〇 |  |  |  |  | COSM13556 | c.2253_2276del |
| exon20 | p.A767_V769dup | 〇 | [1.7] | 〇 | [11.61] |  | COSM12376 | c.2300_2308dup |
|  | p.A767_V769dup | 〇 |  |  |  |  | COSM13558 | c.2309_2310delinsCCAGC GTGGAT |
|  | p.D770_N771insG | 〇 | [1.3] | 〇 | [4.91] |  | COSM12378 | c.2310_2311insGGT |
|  | p.S768_D770dup | 〇 |  |  |  |  | COSM13428 | c.2303_2311dup |
|  | p.H773dup | 〇 | [6.8] | 〇 | [2.40] |  | COSM12377 | c.2317_2319dup |
|  | p.S768I | 〇 | [1.3] | 〇 | [7.66] | 〇 | COSM6241 | c.2303G>T |
|  | p.T790M | 〇 | [2.4] | 〇 | [9.72] | 〇 | COSM6240 | c.2369C>T |
| exon21 | p.L858M |  |  |  |  | 〇 | COSM12366 | c.2572C>A |
|  | p.L858R | 〇 | [4.2] | 〇 | [5.94] | 〇 | COSM6224 | c.2573T>G |
|  | p.L858R | 〇 |  |  |  |  | COSM12429 | c.2573_2574delinsGT |
|  | p.L861Q | 〇 | [2.2] | 〇 | [2.22] | 〇 | COSM6213 | c.2582T>A |
|  | p.L861R |  |  |  |  | 〇 | COSM12374 | c.2582T>G |

Cobas V2: cobas EGFR Mutation Test v2, Therascreen: therascreen EGFR RGQ PCR Kit, Oncomine: Oncomine Dx Target Test, LOD: limit of detection
